# American Institute of Homœopathy

**Published:** 1899-05

**Authors:** J. B. Garrison

**Affiliations:** Chairman Transportation Committee


### AMERICAN INSTITUTE OF HOMŒOPATHY.

### NOTICE.

A reduction of fare and one-third for the round trip has been granted by the Trunk Line Association for those attending the meeting of the Institute at Atlantic City, N. J., in June, on the certificate plan. Tickets will be on sale from June 15th to 21st inclusive. Full fare must be paid for the going ticket, and a certificate—which is prepared by the railroads for the purpose—is given to the purchaser. These certificates are not kept at all local stations, but if not, the agent will sell a ticket to the nearest station where they are kept, and the through ticket and certificate be procured there. Be sure to get the certificate, for without it, properly signed and viséed, the reduction on the return trip cannot be taken advantage of.

As soon as the place of meeting is reached the certificates should be handed to the Chairman of the Transportation Committee, who will sign them and have them viséed by the special agent, and they will then be ready for use when the time comes to return home. The limit for the return expires on June 28th.

No refund of fare will be made on account of any person failing to obtain a certificate.

Fraternally yours,

J. B. GARRISON, M. D.,
*Chairman Transportation Committee.*

